# Success of non‐surgical periodontal therapy in adult periodontitis patients: A retrospective analysis

**DOI:** 10.1111/idh.12399

**Published:** 2019-05-16

**Authors:** G.A. (Fridus) Van der Weijden, Gijs J. Dekkers, Dagmar E. Slot

**Affiliations:** ^1^ Department of Periodontology, Academic Centre for Dentistry Amsterdam (ACTA) University of Amsterdam and Vrije Universiteit Amsterdam Amsterdam The Netherlands; ^2^ Clinic for Periodontology Utrecht Utrecht The Netherlands

**Keywords:** adult periodontitis, bleeding on probing, long‐term follow‐up, probing pocket depth, risk factors, smoking, supportive periodontal treatment, treatment

## Abstract

**Objective:**

To evaluate the results of active non‐surgical treatment in patients diagnosed with adult periodontitis treated in a specialized clinic for periodontology.

**Material & Methods:**

In total, 1182 patients with adult periodontitis received active non‐surgical therapy, which involved professional oral hygiene instruction, scaling and root planing, supragingival polishing and elective systemic antimicrobial medication. The results of this therapy were based on a full‐mouth periodontal chart as assessed at the time of evaluation. Successful treatment as periodontal pocket depth (PPD) ≤5 mm was the main outcome parameter with bleeding on pocket probing as secondary outcome. Patient‐related factors such as smoking and severity of periodontitis at baseline and site‐related factors such as tooth type, furcation involvement and endodontic treatment were analysed. Possible relations with assessed parameters and the success of active periodontal therapy were evaluated.

**Results:**

Overall 39% of the patients reached the successful treatment objective and a mean bleeding on pocket probing tendency of 14%. Treatment success appeared to be dependent on tooth type where the results at single‐rooted front teeth (85%) and premolar teeth (78%) were more successful than at molar teeth (47%). Analysis revealed that in 55% of the cases furcation involvement at molars was associated with the absence of success. Endodontic treatment was associated with absence of success in 8%‐11% of the cases. Smoking negatively influences successful treatment outcome (*P* < 0.001).

**Conclusion:**

Active non‐surgical periodontal therapy in patients with adult periodontitis resulted in approximately one third of the cases in the success endpoint of PPD ≤ 5mm. Sub‐analysis showed that the outcome appeared to be dependent on tooth type, furcation involvement, severity of periodontal disease at intake and smoking status.

## 1 INTRODUCTION

The goal of periodontal therapy is to preserve, improve and maintain the natural dentition.^1^ Therapy includes manual, sonic and/or ultrasonic instrumentation in conjunction with supragingival plaque control.^2^ A systematic review of the literature evaluated the effect of subgingival debridement in terms of bleeding on probing, pocket depth and probing attachment level in patients with chronic periodontitis. Subgingival debridement was found to be an effective treatment in reducing probing pocket depth and improving the clinical attachment level.^3^ Treatment results in a pocket depth reduction due to recession of the gingiva and gain in clinical attachment level. When a pocket depth smaller or equal to 5mm is reached, the treatment can be considered successful.^4^ Various factors have an impact on the success of the active non‐surgical periodontal therapy.^5^

Patient‐associated factors such as severity of the disease and smoking status have a negative effect on periodontal therapy.^6^ Site‐specific factors, for example tooth type and endodontic treatment, may also have an impact on the outcome. With respect to tooth type, there is a difference between single‐rooted and multi‐rooted teeth with possible furcation involvement, which is a complication for the successful treatment of molars.^7^

The aim of the present study was to evaluate the success of active non‐surgical periodontal therapy in patients with adult periodontitis. Successful treatment was considered to be an intra‐oral condition with residual pockets not deeper than 5mm at the point of evaluation.

## 2 MATERIAL & METHODS

This report was prepared according to the guidelines suggested by the STROBE^8^, ^9^ and RECORD^10^, ^11^ checklists. These checklists recommend items that should be included in reports of observational studies and studies using routinely collected observational data. All data were procured retrospectively from the treatment records. Involved patients had provided permission in advance that their treatment outcome could be used for analysis. Approval by the Institutional Review Board of the Academic Centre for Dentistry Amsterdam (ACTA) was provided for this study under number 2017026.

### 2.1 Subjects

Patients involved in the study had been referred because of perio‐dontal problems by their general dentist. They were treated within 12 months following their intake appointment at the specialist Clinic for Periodontology, Utrecht, The Netherlands, from 2013 up to 2016. The clinical diagnosis before active periodontal treatment was moderate‐to‐severe adult periodontitis.^12^, ^13^ The first appointment included intra‐ and extra‐oral assessment, full‐mouth periodontal charting and a complete set of radiographs. The parameters that were collected included probing pocket depth (PPD; measurements were rounded off to the nearest millimetre), bleeding on pocket probing (BOPP scored as absent or present) and furcation involvement (using a PQ0W6 pocket probe and PQZNM furcation probe, Hu‐Friedy, Hu‐Friedy^®^ Inc). PPD and BOPP were recorded at six sites (mesio‐buccal, buccal, disto‐buccal, mesio‐lingual, lingual and disto‐lingual). Based upon these parameters, in relation to age (>35 years old) and in combination with the information as obtained from the radiographs the responsible periodontist came to the clinical diagnosis of (semi) generalized adult periodontitis.^12^, ^13^

Following the intake appointment, the active phase of non‐surgical periodontal therapy was started. Dental hygienists were responsible for the professional tooth debridement and oral hygiene instructions. All patients received 2 to 5 one‐hour sessions of thorough supra‐ and subgingival scaling and root planing of all teeth involved. If necessary, because of individual needs of the patient, this was performed under local anaesthesia. Elective systemic antimicrobial medication consisting of a combination of amoxicillin (375 mg) and metronidazole (250 mg) three times daily for seven days was provided after the treatment sessions at the indication of the periodontist responsible for the treatment. For instrumentation, a piezoelectric ultrasonic unit (Piezon Master, EMS^®^) at a moderate setting and with the appropriate tips for initial therapy (A, P, PS, PL1‐5, EMS^®^) was used. In addition, where deemed appropriate by the dental care professional, hand instruments were utilized (204SD, 12/13 11/14 Hu‐Friedy; Hu‐Friedy^®^ Inc). At each appointment, the level of oral hygiene of the patients was evaluated and if necessary reinforced in case of inadequate plaque control. Patients were scheduled for the interim evaluation, about 6 weeks later by the same dental hygienist. At this appointment, the oral hygiene of the patients was reinforced if after disclosing remaining plaque was made visible. Subgingival debridement was carried out where necessary (ie, PPD > 3mm), and a professional prophylaxis was provided. Approximately two and a half months later, a final evaluation was carried out and PPD, BOPP and furcation involvement were assessed again as described above by the same periodontist.

### 2.2 Data extraction and analysis

The summary data as used for this study were extracted from the patients' files by the periodontist who was responsible for the provided therapy and entered anonymously into a computer database (Microsoft Excel). Data extracted were age, bone loss ≥50%,^12^, ^13^ presence of pockets ≥9mm,^14^ smoking status at intake and the absence or presence of a residual pocket depth at the evaluation appointment not deeper than 5 mm. The latter was also separated by tooth type being front, premolar and molar teeth. The percentage of endodontic treatment was recorded in relation to each of the three tooth types, and for premolars and molars, additionally the presence of furcation involvement was recorded. Furthermore, the percentage BOPP at the evaluation appointment was extracted.

Means, percentages and standard deviations were calculated using Microsoft Excel. Values were mostly ordinal (yes/no), and with the use of Microsoft Excel, crosstabs have been drawn up. Data were entered in SPSS Statistics (Version 24; IBM Corp. Released 2016. IBM SPSS Statistics for Macintosh, Version 24.0.; IBM Corp.). Normal distribution of BOPP was tested by Kolmogorov‐Smirnov and Shapiro‐Wilk. Accordingly, statistical analyses were performed using the chi‐square test and the independent‐samples *t* test. *P*‐values of < 0.05 were considered to be an indicator of statistical significance.

## 3 RESULTS

### 3.1 Demographics

In total 1182 patients included in this study were treated for adult periodontitis between 2013 and 2016. The mean age at intake was 52.6 ± 9.8 years (range 36‐86), and 28.6% of the patients were smokers. The average time of treatment between intake and evaluation was 7.3 ± 1.9 months (range 3‐12).

### 3.2 Treatment success

In total, 39% of the patients ended with pockets not deeper than 5 mm at the evaluation appointment. The success at the front teeth was higher (85%) than at molar teeth (47%). At evaluation, the mean percentage of BOPP at patient level was 13.9 ± 11.1 (Table 1). This was not normally distributed (*P* > 0.0001), and the median BOPP was 11%. If, as proposed by Chapple et al,^15^ a threshold for bleeding on probing of <10% is used for defining a clinical case of health, 44% (n = 515) could be considered as a successfully treated periodontitis patient. When ≤5 mm probing pocket depth is combined with <10% BOPP only in 19% of the patients (n = 226), success was achieved.

**Table 1:** Success of active non‐surgical periodontal treatment N = 1182 as assessed at the evaluation appointment defined as no residual pockets deeper than 5 mm overall separated by tooth type. In addition, the mean percentage of sites that showed bleeding on pocket probing (BOPP) is given

Parameter	Mean (%)	Median	Normal distribution test^a^
Overall success	39		
Success at front teeth	85		
Success at premolars	78		
Success at molars	47		
% BOPP	14	11%	No
<10% BOPP	44		
PPD ≤ 5 mm & <10% BOPP	19		

a Kolmogorov‐Smirnov and Shapiro‐Wilk test.

### 3.3 Non‐treatment success

If the treatment outcome was not successful, that is the presence of residual pockets deeper than 5 mm, tooth‐related factors were evaluated. Endodontic treatment was associated with the absence of success ranging from 8% to 11% of the cases. At the premolar teeth in 10% of the patients, the presence of pockets deeper than 5 mm was associated with furcation involvement. In molar teeth, this was 55% (Table 2).

**Table 2:** Tooth‐related factors in relation to the presence of residual pockets >5 mm at the evaluation appointment separated by tooth type by the number of patients

	Endodontically treated	Furcation involvement
Front teeth (N = 175)	11%	Not applicable
Premolars (N = 264)	11%	10%
Molars (N = 620)	8%	55%

The level of success was associated with the severity of periodontal disease at intake (*P* < 0.001; Table 3a,b). In patients treated successfully, 26% had pockets ≥9 mm at intake as compared to 55% in those that were unsuccessful. Reversely successful treatment was associated with 74% of the patients with pockets <9 mm at intake as compared to 45% in those without success. This difference was significant (*P* < 0.001; Table 3a). Similarly in 80% of the patients without success, the severity of bone loss at intake was higher (as defined by the presence of >50% bone loss). In 49% of the patients treated successfully, the bone loss at intake was ≤50% (*P* < 0.001; Table 3b). At a patient level, the severity of the disease at the intake appointment as assessed based on the presence of pockets ≥9 mm was not associated with the smoking status (*P* = 0.334; Table 3a). There was however a significant relationship between smoking status and severity of bone loss at intake (Table 3b). In smokers, the percentage of patients with bone loss >50% was 77.5% as compared to 66% for non‐smokers, which was a significant difference (*P* < 0.001; Table 3b).

**Table 3:** Severity of disease at intake (a: yes or no pockets ≥9 mm and b: bone loss ≤ or >50%) in relation to the smoking status and absence or presence of residual pockets >5 mm at the evaluation appointment

	Pockets at intake <9 mm (N = 666)	Pockets at intake ≥9 mm (N = 516)	*P*‐value
(a)			
Non‐smoker N = 844	57%	43%	0.334^a^
Smoker N = 338	54%	46%	
Residual pockets ≤5 mm N = 455	74%	26%	<0.001^a^
Residual pockets >5 mm N = 727	45%	55%	

	*≤*50% Bone loss (N = 387)	>50% Bone loss (N = 868)	*P*‐value
(b)			
Non‐smoker N = 844	34%	66%	<0.001^a^
Smoker N = 338	22.5%	77.5%	
Residual pockets ≤5 mm N = 455	49%	51%	<0.001^a^
Residual pockets >5 mm N = 727	20%	80%	

a Chi‐square test was used for the statistical analysis.

At a patient level, non‐smoking was associated with a higher percentage of success than outcome of treatment in patients that did smoke (Table 4). In smokers, treatment in 71% of the cases was unsuccessful. In non‐smokers, treatment was successful in 43% of the patients and unsuccessful in 57%. Non‐smokers therefore showed a significantly better treatment success than smokers (*P* < 0.001). The mean level of BOPP in smokers (15%) at the evaluation appointment was significantly different among smokers and non‐smokers (13%) (*P* = 0.004).

**Table 4:** Smoking status in relation to successful therapy and the mean percentage (SD) and median BOPP

	Residual pockets ≤ 5 mm N = 455	Residual pockets > 5 mm N = 727	BOPP N = 1182	
	Mean (SD)	Mean (SD)	Mean (SD)	Median
Smoker (N = 338)	28%	71%	15% (11)	12
Non‐smoker (N = 884)	43%	57%	13% (11)	11
*P*‐value	<0.001^a^		NA	<0.004^b^

Abbreviation(s): NA, not applicable.

a Chi‐square test was used for the statistical analysis.

b Mann‐Whitney *U* test.

## 4 DISCUSSION

### 4.1 Summary of our findings

The purpose of this retrospective study was to evaluate successfulness of active non‐surgical therapy in adult periodontitis patients. These results represent real‐life situations of a specialized clinic restricted to periodontal therapy. Overall 39% of the patients finished with the predefined level of success being overall with pockets ≤5 mm. Treatment success was more frequently obtained in the front teeth region (85%) and the premolar region (78%). Smoking status, severity of disease and molar furcation involvement appeared to be factors that negatively interfered with success.

### 4.2 Success percentage

The prediction of success of periodontal treatment would be of great benefit for clinicians treating cases of periodontitis, as well as for the patients to allow them to take informed decisions. Conclusions regarding the effect of periodontal therapy are based on the definition of treatment response. In this study, it was chosen to define pockets ≤5 mm at evaluation as “success” of treatment based on the study of Badersten et al.^4^ Other authors have selected other criteria, such as pockets ≤4 mm,^16^ elimination of pockets >3 mm,^17^ elimination of pockets >6 mm^18^, ^19^ or ≤4 sites with PPD ≥ 5 mm.^20^, ^21^ This ambiguity makes comparison between the various studies with different criteria not feasible. The threshold in the present study (PPD ≤ 5 mm) is on the high end of criteria used. Given that in this sample, with a large number of patients being treated in a practice restricted to periodontology, only 39% “success” is obtained, indicates that criteria that are more strict (f.i. ≤4 mm) may be less practical as “endpoint” and could lead to over‐treatment. If on top of PPD ≤ 5 mm also the criterion <10% bleeding on probing is used (see Table 1), as emerges from the consensus statement of the 2017 World Workshop on the Classification of Periodontal Disease,^15^ only 19% of the cases could be considered successfully treated. However, this still does not take into account the more strict threshold of ≤4 mm for defining a clinical case of health in successfully treated periodontitis patients.

Some authors^22^, ^23^ have suggested that “true endpoints” such as “tooth loss” should be used to assess periodontal treatment response. Other studies however use “surrogate endpoints” which include different clinical expressions such as probing pocket depth and bleeding scores.^16^ The American Academy of Periodontology describes in their statement^24^ on comprehensive periodontal therapy; the defined outcome of periodontal therapy to be a significant reduction of clinical signs of gingival inflammation, which also includes probing pocket depth. Based on our definition, the success rate in the present study was 39%, which implies that in 61% the goal was not reached. Of course, the effect of treatment may improve further if a period of maintenance is offered^25^, ^26^ or that additional periodontal therapy such as flap surgery is provided. However, the overall success of 39% from clinical perspective could be considered disappointing. This success percentage is calculated as an overall success rate including all tooth types (front, premolars and molars). Consequently if in one of the tooth types the treatment is not successful (residual pocket depth >5 mm at evaluation) than the treatment is considered to be unsuccessful. On the other hand, if one considers the results in relation to tooth type a higher success rate can be predicted in single‐rooted teeth. Other researchers have also pointed out that if a satisfactory treatment result is obtained the number of successfully treated individuals is much lower than the successfully treated sites. Success rates at site level are higher than the overall success rate due to the fact that many subjects exhibited a mixture of both diseased and healthy sites.^26^ The results of a clinical study with a follow‐up of 36 months after active therapy show that based on a construction of clinical parameters to define success 26% of the individuals exhibited “perfect” periodontal health and were considered to be successfully treated.^26^ In the light of this, the success rate of 39% in the present study could also be seen as an acceptable success.

### 4.3 Relation between tooth type and the effect of active non‐surgical periodontal therapy

The results of the present study show a higher percentage of success in single‐rooted teeth (front teeth: 85%) as compared to molar teeth (47%) (Table 1). This is in agreement with a study where changes in clinical parameters in single‐ and multi‐rooted teeth were evaluated. More positive outcomes were observed by single‐ than by multi‐rooted teeth with respect to the change of initial probing depth to the probing depth at evaluation.^27^ A comparable observation was done by Loos et al^28^ who found that molar furcation sites responded less favourably to periodontal therapy compared to non‐molar sites and molar flat‐surface sites of similar probing depth. Also, other authors have observed that multi‐rooted teeth show less favourable probing pocket depth reduction than single‐rooted teeth.^29^ Possible reason for this is that multi‐rooted teeth can show difficulties in treatment of the periodontal infection due to local anatomical conditions.^30^ The difference in treatment success with respect to tooth type is nicely summarized by Ghiai & Bissada^31^ who state that it is more difficult to predict the outcome correctly of multi‐rooted teeth than single‐rooted ones. Multi‐rooted teeth should also be accounted as a risk factor for possible failure of the periodontal therapy. Other literature states that non‐surgical periodontal therapy is effective and reductions in bleeding on pocket probing and pocket depth are reached in single‐ and in multi‐rooted teeth.^5^, ^32^ Although both tooth types react to the periodontal therapy, single‐rooted teeth showed better treatment results than multi‐rooted teeth. However, there seem to be more site‐associated factors influencing treatment outcome besides tooth type.^5^

One of these influencers is furcation involvement where outcomes from clinical trials showed that molars with furcation involvement responded less favourably to non‐surgical therapy as compared to molars without furcation involvement.^28^, ^33^ Complete debridement of molars will be rarely obtained because of the complex morphology in the furcation area.^34^, ^35^ Therefore, non‐surgical therapy is recommended for teeth with shallow furcation defects (furcation not probeable or the root trunk coronal to the furcation entrance probeable). Non‐surgical periodontal therapy of advanced furcation involvement (furcation entrance probeable >3 mm in horizontal direction or entrance is “through‐and‐through”) usually leads to disease progression in the furcation area with a risk of eventual loss of teeth.^37^ A wide range of alternative treatment modalities have been proposed in the literature based on the depth of the furcation involvement.^38^ A systematic review which has evaluated various therapeutic approaches followed by a period of maintenance care indicates a good survival rate of multi‐rooted teeth with furcation involvement.^39^

### 4.4 Effect of endodontic treated teeth on active non‐surgical therapy

At a site level, endodontic treatment may have an effect on the outcome of periodontal therapy. Patients with teeth that have been endodontically treated show more bone loss compared to contra‐lateral teeth without an endodontic treatment.^40^ In sites with greater bone loss, a poorer treatment response has been reported regarding probing pocket depth reduction.^5^ Also, periodontal disease (pockets > 5 mm) around endodontically treated teeth appears to be an extra risk factor, which can affect tooth survival.^41^, ^42^ In the present study, patients that did not have a successful treatment response had endodontic treatment, which varied between 8% and 11% dependent on tooth type (Table 2). This therefore did not provide a clear explanation why treatment was unsuccessful. Concern about endodontic treatment with respect to treatment success may also be less because Pretzl et al^43^ have shown that it is feasible to retain endodontically treated teeth in periodontitis patients in combination with active and supportive periodontal therapy for more than 10 years. However, after active periodontal therapy, endodontic treatment appeared to be a strong risk factor for tooth loss of molars.

### 4.5 Effect smoking status and non‐surgical periodontal therapy

Smoking has proven to be a major risk factor in the prevalence, extent and severity of periodontitis.^44^, ^45^ Van der Weijden et al^48^ reported that cigarette smoking is a factor associated with deeper periodontal pockets. As is apparent from Table 3 in the present study, no significant difference was found between smokers and non‐smokers with respect to the presence of initially deep pockets (>9 mm; *P* = 0.334) (Table 3a). The reason for this is unclear but may be related to the relatively high cut‐off of 9 mm that was chosen to discern between moderate and deep pockets.

Duane^49^ shows that there is a strong association between chronic smoking and bone loss which is in agreement with the present study. Table 3b shows that smokers presented more often with bone loss (>50%) than non‐smokers (*P* < 0.001). Several studies have also shown that smokers have a poorer response to non‐surgical periodontal therapy than non‐smokers.^50^, ^51^ This also is in agreement with the results of the present study where treatment in non‐smokers was more successful than in smokers (*P* < 0.001, Table 4). Similarly, Renvert et al (1998) 52 have shown that the treatment response in non‐smokers was better than in smokers. They observed following non‐surgical periodontal therapy in non‐smokers a pocket depth reduction of 2.5mm as compared to a reduction in smokers of 1.9mm. Other authors have observed that smoking cessation promoted an additional benefit in the reduction of probing pocket depth.^53^, ^54^ With respect to bleeding scores, Renvert et al^52^ observed a higher level of residual inflammation with a full‐mouth bleeding score in smokers of 37% as compared to 23% in non‐smokers. The present study did not find such a large difference in mean percentage bleeding scores between smokers (15%) and non‐smokers (13%) (Table 4). However, smoking has a detrimental effect on the incidence and progression of periodontitis. Tobacco smoking, therefore, is important information that should be assessed along with other risk factors for periodontitis.^55^

### 4.6 Severity of periodontitis and treatment success

According to the literature, all severities of periodontitis can be treated well and should show a positive effect following the non‐surgical periodontal therapy.^56^, ^57^ In the present study, severity of periodontitis is determined by bone loss >50% (yes/no) and initial pocket depth ≥9 mm (yes/no). These two factors have a significant negative impact on the treatment outcome (Tables 3 and 4). Some authors have provided guidelines for the daily practice to address the “critical probing depth”.^58^ In sites with initial probing depths above the “critical probing depth” value, a better result occurred following periodontal surgery than following non‐surgical periodontal therapy.^59^ Their data also disclosed that the level of oral hygiene maintained by the patients during healing and maintenance was more critical for the resulting probing depths and attachment levels than the mode of initial therapy used.

### 4.7 Treatment facility

The present study is to some extent limited by the records of the Clinic for Periodontology, which rely on data from clinicians’ and patient‐reported data regarding smoking habits. It has however been shown that treatment provided in the private periodontal clinic showed significantly less progression of periodontitis and tooth loss as compared to treated in an academic setting.^60^ A recent retrospective study evaluated the effect of supportive periodontal treatment from the same periodontal practice as the present study. It showed that after 10 years on average 2.6 teeth were lost.^61^ Based on a recent systematic review, it appears that specialist periodontal maintenance is effective in sustaining periodontal stability following active specialist intervention. There is some evidence that primary dental care provides the same level of care. The limited comparative data available suggest that outcomes could be slightly worse in primary dental care.^62^

### 4.8 Limitations

Baseline pocket depth was not entered into the database. In retrospect, more details on baseline disease characteristics would have provided more insight into the population of this study. However, one should realize that the participants were all patients that had been referred by their general dentist for periodontal disease. This was disease that was beyond the control of what they considered could be effectively treated in general practice. This aspect provides a reflection of the population under investigation. Also, Van der Velden (2005) defined periodontitis as the presence of inflamed pathological pockets ≥4 mm deep in conjunction with attachment loss. The classification (semi) generalized “adult” periodontitis was based on the extent of the disease (at least 8 teeth involved) and the participants’ age. “Moderate to severe” concerned bone loss >1/3 or >1/2 of the root length and attachment loss 4‐5 mm or ≥6 mm, respectively.

It was also not recorded how many cigarettes each day were smoked by the patients. Further research needs to determine whether there is a correlation between the amount of cigarettes smoked and the success of periodontal therapy. In addition, there were also no data if non‐smoking patients were former smokers or may have started smoking during the treatment. According to the literature, smoking is also associated with the development of systemic diseases, such as diabetes mellitus. Adults with diabetes also have an increased risk of developing periodontitis than those without.^63^ This aspect was not noted in the database.

Patients in the study also had been treated endodontically. It is not recorded in the database if these patients still had peri‐apical radiolucencies. Also, furcation involvement was only scored as being present or not by the degree of furcation accessibility.

A number of patients received elective systemic antimicrobial medication at the indication of the periodontists in charge. The reason for this was however not recorded. This may have had a positive impact on the treatment outcome.^64^

Many determinants of treatment outcomes from the present analysis have already been taken into consideration separately by previous authors. Therefore, the originality of the present study could be considered as limited. However, never before were these determinants analysed in one study with such a high number of patients from a regular periodontal practice.

The present study retrospectively analysed data from patient records that involved periodontal treatment in the period of 2013‐2016. At that time, patients were classified at intake according to the definition of Van der Velden.^12^, ^13^ Based on age, those patients that were classified as “adult periodontitis” were selected for the present evaluation of treatment success. Given the new classification which was recently introduced,^65^ patients would now be classified as having “periodontitis”.

Lang & Bartold^66^ state in their review concerning treatment targets for a diseased periodontium that mean values of PPD are not an adequate predictor for sites that may become reinfected and undergo recurrent disease. They suggest that PPD must be considered in conjunction with other important clinical parameters such as bleeding on pocket probing (BOPP), as well as modifying and predisposing factors. In the present study, success was evaluated at patient level and was defined as the absence of pockets >5 mm. Absence of BOPP was not taken into account and could be the subject of future studies.

The number of teeth present at intake and those extracted during non‐surgical periodontal therapy were not taken from the individual patient records. This would have provided related information.

The level of oral hygiene is a factor related to presence of residual pockets after therapy. These data were not analysed. However, individual oral hygiene instructions and reinforcement were part of the treatment protocol.

## 5 CONCLUSION

This present study shows that active non‐surgical periodontal therapy in patients with adult periodontitis resulted in approximately one third of the cases in the success endpoint of no pockets deeper than 5 mm. Sub‐analysis showed that the outcome appeared to be dependent on different factors, such as tooth type, furcation involvement and smoking. Treatment success was higher at single‐rooted teeth than at molar teeth, especially in those with furcation involvement. Success rate was also related to the severity of periodontal disease at intake and to the smoking status.

## 6 CLINICAL RELEVANCE

### 6.1 Scientific rationale for the study

Periodontitis is primarily treated with non‐surgical periodontal therapy. When a probing pocket depth (PPD) ≤5 mm is reached, the treatment can be considered as successful. In this retrospective study, the results of active non‐surgical periodontal therapy in a clinic restricted to periodontology are evaluated.

### 6.2 Principal findings

Active non‐surgical periodontal therapy in patients with adult periodontitis resulted in approximately one third of the cases in “success” (PPD ≤ 5 mm).

### 6.3 Practical implications

Success following active non‐surgical periodontal therapy in patients with adult periodontitis is limited. Especially in molar teeth, it is difficult to reach pockets ≤ 5 mm. Dental care professionals should consider this when they estimate the prognosis of teeth and molars.

## CONFLICT OF INTEREST

The authors declare that they have no conflicts of interest.

## AUTHOR CONTRIBUTION

All authors gave final approval and agreed to be accountable for all aspects of work ensuring integrity and accuracy. GAW contributed to conception and design, analysis and interpretation, and drafted the manuscript. GJD contributed to analysis and interpretation, and drafted the manuscript. DES contributed to the design, analysis and interpretation, and critically revised the manuscript.

## References

[1] Slot DE , Van der Weijden FA . Current evidence on lasers as adjuncts to nonsurgical periodontal therapy. Intern J EBP Dent Hyg. 2015;1:92‐97.

[2] Cobb CM . Clinical significance of non‐surgical periodontal therapy: an evidence‐based perspective of scaling and rootplaning. J Clin Periodontol. 2002;2:6‐16. 12010523

[3] Van der Weijden GA , Timmerman MF . A systematic review on the clinical efficacy of subgingival debridement in the treatment of chronic periodontitis. J Clin Periodontol. 2002;29:55‐71. 12787207 10.1034/j.1600-051x.29.s3.3.x

[4] Badersten A , Nilveus R , Egelberg J . Effect of nonsurgical periodontal therapy. II. Severely advanced periodontitis. J Clin Periodontol. 1984;11:63‐76. 6363463 10.1111/j.1600-051x.1984.tb01309.x

[5] Kim TS , Schenk A , Lungeanu D , Reitmeir P , Eickholz P . Nonsurgical and surgical periodontal therapy in single‐rooted teeth. Clin Oral Investig. 2007;11:391‐399. 10.1007/s00784-007-0144-x PMC2134843 17690922

[6] Bunaes DF , Lie SA , Astrom AN , Mustafa K , Leknes KN . Site‐specific treatment outcome in smokers following 12 months of supportive periodontal therapy. J Clin Periodontol. 2016;43:1086‐1093. 27554463 10.1111/jcpe.12619 PMC5132109

[7] Gusmão ES , Picarte AC , Ben Barbose MB , Rösing CK , Cimoes R . Correlation between clinical and radiographic findings on the occurrence of furcation involvement in patients with periodontitis. Indian J Dent Res. 2014;25:572‐575. 25511053 10.4103/0970-9290.147086

[8] von Elm E , Altman DG , Egger M , et al. The Strengthening the Reporting of Observational Studies in Epidemiology (STROBE) statement: guidelines for reporting observational studies. J Clin Epidemiol. 2008;61:344‐349. 18313558 10.1016/j.jclinepi.2007.11.008

[9] Webdocument. STROBE‐statement; https://www.strobe-statement.org/index.php?xml:id=strobe-home. Accessed September 25, 2018.

[10] Nicholls SG , Quach P , von Elm E , et al. The reporting of studies conducted using observational routinely‐collected health data (RECORD) statement: methods for arriving at consensus and developing reporting guidelines. PLoS ONE. 2015;10(5):e0125620. 25965407 10.1371/journal.pone.0125620 PMC4428635

[11] Webdocument. RECORD‐statement; http://www.record-statement.org/. Accessed September 25, 2018.

[12] Van der Velden U . Purpose and problems of periodontal disease classification. Periodontol. 2000;2005(39):13‐21. 10.1111/j.1600-0757.2005.00127.x 16135060

[13] Van der Velden U . Diagnosis of periodontitis. J Clin Periodontol. 2000;27:960‐961. 11140565 10.1034/j.1600-051x.2000.027012960.x

[14] Joyston‐Bechal S , Smales FC , Duckworth R . Effect of metronidazole on chronic periodontal disease in subjects using a topically applied chlorhexidine gel. J Clin Periodontol. 1984;11(1):53‐62. 6363462 10.1111/j.1600-051x.1984.tb01308.x

[15] Chapple I , Mealey BL , Van Dyke TE , et al. Periodontal health and gingival diseases and conditions on an intact and a reduced periodontium: consensus report of workgroup 1 of the 2017 World Workshop on the classification of periodontal and peri‐implant diseases and conditions. J Clin Periodontol. 2018;45(Suppl 20):S68‐S77. 29926499 10.1111/jcpe.12940

[16] Baelum V , Lopéz R . Defining and predicting outcomes of non‐surgical periodontal treatment: a 1‐yr follow‐up study. Eur J Oral Sci. 2016;124:33‐44. 26714428 10.1111/eos.12240

[17] Kolakovic M , Held U , Schmidlin PR , Sahrmann P . An estimate of pocket closure and avoided needs of surgery after scaling and root planing with systemic antibiotics: a systematic review. BMC Oral Health. 2014;14:159. 25529408 10.1186/1472-6831-14-159 PMC4531502

[18] Preus HR , Gunleiksrud TM , Sandvik L , Gjermo P , Baelum V . A randomized, double‐masked clinical trial comparing four periodontitis treatment strategies: 1‐year clinical results. J Periodontol. 2013;84:1075‐1086. 23106511 10.1902/jop.2012.120400

[19] Sanz M , Bäumer A , Buduneli N , et al. Effect of professional mechanical plaque removal on secondary prevention of prevention of periodontitis and the complications of gingival and periodontal preventive measures: consensus report of group 4 of the 11th European Workshop on Periodontology on effective prevention of periodontal and peri‐implant diseases. J Clin Periodontol. 2015;42:214‐220. 10.1111/jcpe.12367 25626357

[20] Mestnik MJ , Feres M , Figueiredo LC , et al. The effects of adjunctive metronidazole plus amoxicillin in the treatment of generalized aggressive periodontitis: a 1‐year double‐blinded, placebo‐controlled, randomized clinical trial. J Clin Periodontol. 2012;39:955‐961. 22882646 10.1111/j.1600-051X.2012.01932.x

[21] Teughels W , Durukan A , Ozcelik O , Pauwels M , Quirynen M , Haytac MC . Clinical and microbiological effects of *Lactobacillus reuteri* probiotics in the treatment of chronic periodontitis: a randomized placebo‐controlled study. J Clin Periodontol. 2013;40:1025‐1035. 24164569 10.1111/jcpe.12155 PMC3908359

[22] Hujoel PP , DeRouen TA . A survey of endpoints characteristics in periodontal clinical trials published 1988–1992, and implications for future studies. J Periodontol. 1995;22:397‐407. 10.1111/j.1600-051x.1995.tb00167.x 7601922

[23] Hujoel PP . Endpoints in periodontal trials: the need for an evidence‐based research approach. Periodontol 2000. 2004;36(1):196‐204. 15330950 10.1111/j.1600-0757.2004.03681.x

[24] Academy of Periodontology (AAP) . Comprehensive periodontal therapy: a statement by the American Academy of Periodontology. J Periodontol. 2011;82:943‐949. 21721992 10.1902/jop.2011.117001

[25] Badersten A , Nilvéus R , Egelberg J . Effect of nonsurgical periodontal therapy. I. Moderately advanced periodontitis. J Clin Periodontol. 1981;8:57‐72. 6972954 10.1111/j.1600-051x.1981.tb02024.x

[26] Lundgren D , Asklöw B , Thorstensson H , Härefeldt AM . Success rates in periodontal treatment as related to choice of evaluation criteria. J Periodontol. 2001;28:23‐30. 10.1034/j.1600-051x.2001.280104.x 11142663

[27] Meseli SE , Kuru B , Kuru L . Relationships between initial probing depth and changes in the clinical parameters following non‐surgical periodontal treatment in chronic periodontitis. J Istanb Univ Fac Dent. 2017;51:11‐17. 29114425 10.17096/jiufd.40993 PMC5624140

[28] Loos B , Nylund K , Claffey N , Egelberg J . Clinical effects of root debridement in molar and non‐molar teeth. A 2‐year follow‐up. J Periodontol. 1989;16:498‐504. 10.1111/j.1600-051x.1989.tb02326.x 2778083

[29] D'Aiuto F , Ready D , Parkar M , Tonetti MS . Relative contribution of patient‐, tooth‐, and site‐associated variability on the clinical outcomes of subgingival debridement. I. Probing depths. J Periodontol. 2005;76:398‐405. 15857074 10.1902/jop.2005.76.3.398

[30] Ehnevid H , Jansson LE . Effects of furcation involvements on periodontal status and healing in adjacent proximal sites. J Periodontol. 2001;71:871‐876. 10.1902/jop.2001.72.7.871 11495134

[31] Ghiai S , Bissada NF . Prognosis and actual treatment outcome of periodontally involved teeth. Periodontal Clin Investig. 1996;18:7‐11. 9116464

[32] Vouros I , Konstantinidis A , Kirkou‐Bata A . Effect of non‐surgical periodontal therapy in an undergraduate dental clinic. Results one year following treatment. J Biol Buccale. 1992;20:11‐17. 1522081

[33] Nordland P , Garrett S , Kiger R , Vanooteghem R , Hutchens LH , Egelberg J . The effect of plaque control and root debridement in molar teeth. J Clin Periodontol. 1987;14:231‐236. 3294917 10.1111/j.1600-051x.1987.tb00972.x

[34] Matia JI , Bissada NF , Maybury JE , Ricchetti P . Efficiency of scaling of the molar furcation area with and without surgical access. Int J Periodontics Restorative Dent. 1986;6:25‐35. 3542872

[35] Fleischer HC , Mellonig JT , Brayer WK , Gray JL , Barnett JD . Scaling and root planing efficacy in multi‐rooted teeth. J Periodontol. 1989;60:402‐409. 2674398 10.1902/jop.1989.60.7.402

[36] Takacs VJ , Lie T , Perala DG , Adams DF . Efficacy of 5 machining instruments in scaling of molar furcations. J Periodontol. 1993;64:228‐236. 8463946 10.1902/jop.1993.64.3.228

[37] Salvi GE , Mischler DC , Schmidlin K , et al. Risk factors associated with the longevity of multi‐rooted teeth. Long‐term outcomes after active and supportive periodontal therapy. J Clin Periodontol. 2014;41:701‐707. 24766602 10.1111/jcpe.12266

[38] Svärdström G , Wennström JL . Periodontal treatment decisions for molars: an analysis of influencing factors and long‐term outcome. J Periodontol. 2000;71:579‐585. 10807122 10.1902/jop.2000.71.4.579

[39] Huynh‐Ba G , Kuonen P , Hofer D , Schmid J , Lang NP , Salvi GE . The effect of periodontal therapy on the survival rate and incidence of complications of multi‐rooted teeth with furcation involvement after an observation period of at least 5 years: a systematic review. J Clin Periodontol. 2009;36:164‐176. 19207893 10.1111/j.1600-051X.2008.01358.x

[40] Timmerman MF , Van der Weijden GA . Bone level around endodontically treated teeth in periodontitis patients. J Clin Periodontol. 2006;33:620‐625. 16856903 10.1111/j.1600-051X.2006.00958.x

[41] Skupien JA , Opdam NJ , Winnen R , et al. Survival of restored endodontically treated teeth in relation to periodontal status. Braz Dent J. 2016;27:37‐40. 27007343 10.1590/0103-6440201600495

[42] Graetz C , Schützhold S , Plaumann A , et al. Prognostic factors for the loss of molars – an 18‐years retrospective cohort study. J Periodontol. 2015;42:943‐950. 10.1111/jcpe.12460 26399690

[43] Pretzl B , Eichkholz P , Saure D , Pfefferle T , Zeidler A , Dannewitz B . Endodontic status and retention of molars in periodontally treated patients: results after 10 or more years of supportive periodontal therapy. J Clin Periodontol. 2016;43:1116‐1123. 27570936 10.1111/jcpe.12621

[44] Albander JM . Global risk factors and risk indicators for periodontal diseases. Periodontol 2000. 2002;29(1):177‐206. 12102708 10.1034/j.1600-0757.2002.290109.x

[45] Burt B . Research science and therapy committee of the American Academy of Periodontology. Position paper: epidemiology of periodontal diseases. J Periodontol. 2005;76:1406‐1419.** 16101377 10.1902/jop.2005.76.8.1406

[46] Chambrone L , Preshaw PM , Rosa EF , et al. Effects of smoking cessation on the outcomes of non‐surgical periodontal therapy: a systematic review and individual patient data meta‐analysis. J Periodontol. 2013;40:607‐615. 10.1111/jcpe.12106 23590649

[47] Genco RJ , Borgnakke WS . Risk factors for periodontal disease. Periodontol. 2000;2013(62):59‐94. 10.1111/j.1600-0757.2012.00457.x 23574464

[48] Van der Weijden GA , De Slegte C , Timmerman MF , Van der Velden U . Periodontitis in smokers and non‐smokers: intra‐oral distribution of pockets. J Clin Periodontol. 2001;28:955‐960. 11686814 10.1034/j.1600-051x.2001.028010955.x

[49] Duane B . Further evidence that periodontal bone loss increases with smoking and age. Evid Based Dent. 2014;15:72‐73. 25343388 10.1038/sj.ebd.6401038

[50] Labriola A , Needleman I , Moles DR . Systematic review of the effect of smoking on nonsurgical periodontal therapy. Periodontal. 2000;2005(37):124‐137. 10.1111/j.1600-0757.2004.03793.x 15655029

[51] Wan CP , Leung WK , Wong M , et al. Effects of smoking on healing response to non‐surgical periodontal therapy: a multilevel modelling analysis. J Periodontol. 2009;36:229‐239. 10.1111/j.1600-051X.2008.01371.x 19236535

[52] Renvert S , Dahlén G , Wilkström M . The clinical and microbiological effects of non‐surgical periodontal therapy in smokers and non‐smokers. J Clin Periodontol. 1998;25:153‐157. 9495614 10.1111/j.1600-051x.1998.tb02421.x

[53] Preshaw PM , Heasman L , Stacey F , Steen N , McCracken GI , Heasman PA . The effect of quitting smoking on chronic periodontitis. J Clin Periodontol. 2005;32:869‐879. 15998271 10.1111/j.1600-051X.2005.00779.x

[54] Rosa EF , Corraini P , de Carvalho VF , et al. A prospective 12‐month study of the effect of smoking cessation on periodontal clinical parameters. J Periodontol. 2011;38:562‐571. 10.1111/j.1600-051X.2011.01723.x 21488933

[55] Leite F , Nascimento GG , Scheutz F , López R . Effect of smoking on periodontitis: a systematic review and meta‐regression. Am J Prev Med. 2018;54:831‐841. 29656920 10.1016/j.amepre.2018.02.014

[56] Balata ML , Andrade LP , Santos DB , et al. Photodynamic therapy associated with full‐mouth ultrasonic debridement in the treatment of severe chronic periodontitis: a randomized‐controlled clinical trial. J Appl Oral Sci. 2013;21:208‐214. 23739857 10.1590/1678-7757201302366 PMC3881869

[57] Mlachkova AM , Popova CL . Efficiency of non‐surgical periodontal therapy in moderate chronic periodontitis. Folia Med. 2014;56:109‐115. 10.2478/folmed-2014-0016 25181848

[58] Graziani F , Karapetsa D , Mardas N , Leow N , Donos N . Surgical treatment of the residual periodontal pocket. Periodontol. 2000;2018(76):150‐163. 10.1111/prd.12156 29193404

[59] Lindhe J , Socransky SS , Nyman S , Haffajee A , Westfelt E . “Critical probing depths” in periodontal therapy. J Clin Periodontol. 1982;9:323‐336. 6764782 10.1111/j.1600-051x.1982.tb02099.x

[60] Costa FO , Santuchi CC , Lages EJ , et al. Prospective study in periodontal maintenance therapy: comparative analysis between academic and private practices. J Periodontol. 2012;83:301‐311. 21780903 10.1902/jop.2011.110101

[61] De Wet LM , Slot DE , Van der Weijden GA . Supportive periodontal treatment: Pocket depth changes and tooth loss. Int J Dent Hyg. 2018;16:210‐218. 28618120 10.1111/idh.12290

[62] Leavy PG , Robertson DP . Periodontal maintenance following active specialist treatment: should patients stay put or return to primary dental care for continuing care? A comparison of outcomes based on the literature. Int J Dent Hyg. 2018;16:68‐77. 28544259 10.1111/idh.12288

[63] Hong M , Kim HY , Seok H , et al. Prevalence and risk factors of periodontitis among adults with or without diabetes mellitus. Korean J Intern Med. 2016;31:910‐919. 27604799 10.3904/kjim.2016.031 PMC5016291

[64] Zandbergen D , Slot DE , Niederman R , Van der Weijden FA . The concomitant administration of systemic amoxicillin and metronidazole compared to scaling and root planing alone in treating periodontitis: ‐a systematic review‐. BMC Oral Health. 2016;29(16):27. 10.1186/s12903-015-0123-6 PMC4770674 26928597

[65] Tonetti MS , Greenwell H , Kornman KS . Staging and grading of periodontitis: framework and proposal of a new classification and case definition. J Clin Periodontol. 2018;45(Suppl 20):S149‐S161. 29926495 10.1111/jcpe.12945

[66] Lang NP , Bartold PM . Periodontal health. J Clin Periodontol. 2018;45(Suppl 20):S9‐S16. 29926485 10.1111/jcpe.12936

